# B-Cell Receptor Signaling Is Thought to Be a Bridge between Primary Sjogren Syndrome and Diffuse Large B-Cell Lymphoma

**DOI:** 10.3390/ijms24098385

**Published:** 2023-05-07

**Authors:** Leila Mohammadnezhad, Mojtaba Shekarkar Azgomi, Marco Pio La Manna, Giuliana Guggino, Cirino Botta, Francesco Dieli, Nadia Caccamo

**Affiliations:** 1 Central Laboratory of Advanced Diagnosis and Biomedical Research (CLADIBIOR), AOUP Paolo Giaccone, 90127 Palermo, Italy; leila.mohammadnezhad@unipa.it (L.M.); marcopio.lamanna@unipa.it (M.P.L.M.);; 2 Department of Sciences for Health Promotion and Mother-Child Care “G. D’Alessandro”, University of Palermo, 90127 Palermo, Italy; 3 Department of Biomedicine, Neuroscience and Advanced Diagnosis (BIND), University of Palermo, 90127 Palermo, Italy

**Keywords:** primary Sjogren syndrome, non-hodgkins lymphoma, DLBCL, cell signaling, BCR

## Abstract

Primary Sjogren syndrome (pSS) is the second most common autoimmune disorder worldwide, which, in the worst scenario, progresses to Non-Hodgkin Lymphoma (NHL). Despite extensive studies, there is still a lack of knowledge about developing pSS for NHL. This study focused on cells’ signaling in pSS progression to the NHL type of diffuse large B-cell lymphoma (DLBCL). Using bulk RNA and single cell analysis, we found five novel pathologic-independent clusters in DLBCL based on cells’ signaling. B-cell receptor (BCR) signaling was identified as the only enriched signal in DLBCL and pSS peripheral naive B-cells or salivary gland-infiltrated cells. The evaluation of the genes in association with BCR has revealed that targeting *CD79A*, *CD79B*, and *LAMTOR4* as the shared genes can provide novel biomarkers for pSS progression into lymphoma.

## 1. Introduction

pSS is an autoimmune disorder characterized by chronic arthritis along with dry eyes and dry mouth [1]. Based on the serological and genetic characteristics of patients presenting with sicca-related manifestations either alone or in the presence of other systemic autoimmune diseases, the terms primary and secondary Sjogren are coined, respectively [2]. pSS is the second most common systemic autoimmune disease after rheumatoid arthritis [3], with a worldwide distribution that predominantly affects females (female/male ratio 9/1) [4].

Despite being thoroughly investigated, the precise process by which pSS presents and evolves is still unknown. The production of cytokines, chemokines, and adhesion molecules that are dysregulated in response to environmental or hormonal triggers and genetic predispositions may accelerate the evolution of the pSS [5]. The critical biologic phenomena that mark the autoimmune nature of pSS are infiltration of lymphocytes in epithelial tissues and hyperactivation of B-cells in affected patients [6,7]. B-cell participation in pSS pathogenesis is further supported by the disease’s progression to B-cell malignancies, like NHL, the most severe complication associated with pSS [8]. Abnormal intracellular signaling, erratic transcriptional and epigenetic control, and immune evasion are major themes in the pathogenesis of NHL [9]. Abnormal intracellular signaling might lead to an imbalanced survival of B-cells, resulting in their malignant transformation. To understand the pathophysiology of these cells, we have primarily relied on the body of knowledge on normal B-cell biology.

Briefly, B-cell developmental events consist of sequential processes leading to the assembly, expression, and signaling of the BCR and selection through central tolerance [10]. Through the formation of the BCR, the immunoglobulin genes undergo V(D)J recombination in the bone marrow, resulting in the extracellular expression of IgM. During this process, the surface IgM with Igα (CD79a) and Igβ (CD79b) subunits drives survival signals to maturing B-cells [11,12]. Indeed, in secondary lymphoid organs, the recognition of non-self antigen drives the aggregation of the BCR on the plasma membrane, which triggers the activation of the BCR-associated kinases. Two classes of protein tyrosine kinase Src-PTKs (which contain Lyn, Fyn, Lck, and Blk) and the Syk family are associated with the BCR complex. Following BCR activation, Lyn, and other Src-PTKs kinases phosphorylate the immunoreceptor tyrosine-based activation motif (ITAM) in the cytoplasmatic domain of CD79a and CD79b. Phosphorylated ITAM, together with Src-PTKs, activate the Syk family to initiate several signaling cascades. In addition, the BCR provides survival for all mature B-cells either in the presence or absence of antigen through weak “tonic” signals [11,13,14,15]. The binding of BCR with an antigen or cell-autonomous pathways triggers the activation or inactivation of variable signaling pathways, such as the NF-κB, PI3K/mTOR, MAPK, and NFAT pathways [12,16,17]. Thus, BCR signaling is critical in the determination of the B-cell’s fate, while its constitutive and chronic activation might accelerate many autoimmune diseases and/or malignancies. Malignant B-cells frequently exploit normal B-cell signaling pathways, such as NF-κB and PI3K, and orchestrate multiple downstream survival pathways to ensure their development and survival [9,11]. Such events occur in diffuse large B-cell lymphoma (DLBCL), which includes two main subtypes: germinal center B-cell (GCB) and activated B-cell (ABC) lymphoma [18]. Targeting these signals to inhibit abnormal B-cells has always been a source of hope for treating autoimmune and cancer diseases [17,19,20].

The most pSS-associated lymphoma subtype is the marginal zone lymphoma (MZL) of the mucosa-associated lymphoid tissue (MALT) type [21], which normally has a benign clinical course but can transform into an aggressive B-cell lymphoma like DLBCL with a poor prognosis [22]. Lack of sufficient and available MALT data and the possibility of progression from pSS into DLBCL led us to focus on DLBCL subtypes [23]. To the best of our knowledge, there haven’t been many studies on how pSS progresses to DLBCL. For this reason, we investigated B-cell signaling pathways in pSS that, in the worst scenario, drive pSS to progress to DLBCL. To better understand common underlying molecular mechanisms during this progression step, we have taken advantage of in-silico studies from available datasets. Thanks to the widespread use of RNA sequencing (RNA-seq), a meta-analysis can offer an overview of either the enriched signals or the numerous genes that are upregulated/downregulated during these pathways. In this study, we used published data from DLBCL and pSS patients to find novel biomarkers for the hyperactivation of BCR in pSS and lymphoma.

## 2. Results

### 2.1. Development of a BCR Signaling Gene Signature

**Figure 1:**
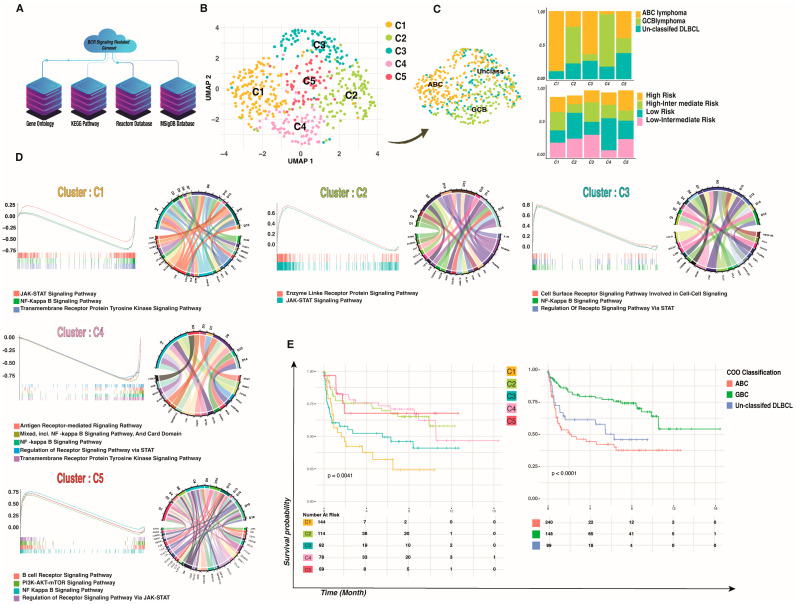
BCR signaling-based clusters in DLBCL. (**A**) Workflow for BCR signaling-based geneset reconstitution that was used as the backbone of the study. (**B**) Five hundred patients were combined and visualized using UMAP association; five novel clusters were detected in DLBCL based on BCR signaling that are independent of IPI and pathologic condition. (**C**) The IPI and old gene expression profiling (GEP) classification of DLBCL that contain two major molecular subtypes according to their COO in each new cluster showed that our clusters are independent from pathologic conditions. (**D**) The enrichment of BCR-signaling genesets in DLBCL novel clusters showed different signaling manners in each cluster; the y axes indicate NES and the x axes indicate enrichment core in each cluster versus other clusters (left to right), while the Circo plots represent the conserved markers belonging to each geneset. (**E**) Survival analysis of DLBCL patients. PFS of patients with DLBCL based on BCR signaling-based clusters and COO classification. A two-tailed *p* < 0.05 was considered statistically significant.

Gene ontology (GO) and the Kyoto Encyclopedia of Genes and Genomes (KEGG) database were used to collect genes or groups of genes/proteins for BCR signaling function analysis. A table that includes all of the gene sets identified through text mining was developed and is reported (Table 1). Initially, this table contained 626 unique genes selected from 16 BCR-related terms. At the retrieval step, we applied the STRING network for the 626 unique proteins to control the correlation of selected genes with BCR signaling, resulting in 612 nodes and 20,095 edges with a default confidence score ≥ 0.4. We used functional enrichment analysis with a false discovery rate (FDR) threshold of 5%, which resulted in a list of 129 statistically significant terms that span six categories: the three GO (GO Biological Process, GO Molecular Function, and GO Cellular Component) and the three KEGG (Pathways, Reactom Database, and MSigDB Dataset) (Figure 1A). We next used the filter functionality to eliminate redundant terms (using the default redundancy cutoff of 0.5), and all signaling pathways containing at least 10 core genes were chosen (Supplementary Data File). As a result, the list was reduced to a more manageable 221 unique enriched terms, which served as the study’s backbone (Supplementary Data File and Table 1). We used generic terms from Table 1 to identify conserved markers, while enriched terms were used for functional enrichment analysis.

### 2.2. A High Multi-Omics Cluster of DLBCL Based on Signaling Genes

Illumina hiSeq RNASeq RNA expression datasets of patients diagnosed with DLBCL and for whom clinical information was available (*n* = 529), consisting of two different projects, were downloaded from the National Cancer Institute (NCI) Center for Cancer Research (CCR) (“NCICCR-DLBCL”, *n* = 481) of The Cancer Genome Atlas (TCGA, https://tcga-data.nci.nih.gov/tcga/, 11 January 2023) (“TCGA-DLBC”, *n* = 48) program. To date, many biomarker discovery studies in DLBCL samples have been enabled by high-throughput and next-generation sequencing (NGS) [24,25], which have been inconsistent because of limitations such as tissue heterogeneity, small sample sizes, and single-cohort techniques.

In our study, we started to analyze single-cell RNA sequencing (scRNA-seq) data in pSS patients using conventional pipelines, but we failed to find any novel putative cancer-specific biomarkers linked to BCR signaling common to those altered in DLBCL. Therefore, we used an innovative multi-omics clustering algorithm at the bulk sequencing level in order to find biomarkers in a large sample cohort of DLBCL that are changing among all samples and then train those biomarkers in a small sample cohort of pSS with a high resolution of gene expression.

To provide a comprehensive context in which to investigate the BCR signaling pathway in DLBCL from the perspective of 626 genes associated with BCR signaling in a data-driven modality and independent of prior assumptions, we employed an in-house developed multi-omics clustering algorithm in single patient resolution to access different DLBCL sub-clusters based on the BCR signaling pathway. After quality control and filtering, a total of 500 patients/transcriptomes were obtained, with a median of 31,250 genes per patient. We first used dimensional reduction and clustering based on the BCR signaling-related geneset to assess patient-to-patient variability and clustering according to the expression of the BCR signaling-related geneset. Next, we used bulk RNA-seq analysis for the total of the previously described 500 samples at single patient resolution, and we identified five different subclusters within the BCR signaling-related clusters (Figure 1B).

Since current standard chemo-immunotherapy does not cure roughly one-third of DLBCL patients [26], patients’ classification based on DLBCL molecular pathogenesis can improve treatment outcomes. As a result, and given the consistency of donors, we first merged and normalized all 500 samples to create a patient-specific reference atlas of DLBCL. Then we performed the correction for batch effects between transcriptional profiles from each patient by matching mutual nearest neighbors [27] prior to principal component analysis (PCA) and visualization via uniform manifold approximation and projection (UMAP). This analysis demonstrated the clustering of patients broadly by BCR-related transcriptome change rather than on patients’ pathological classification, suggesting successful mitigation of batch effects (Supplementary Figure S1). This final single object included two different pathological subtypes and four different International Prognostic Indicators (IPI) (Figure 1C).

We then performed unsupervised graph-based clustering. For each patient, we identified transcriptionally distinct clusters based on their 200-highest marker gene expression profile (Supplementary Data File). Typically, the cell cluster was visualized using UMAP, as shown in Figure 1B. The analysis of the merged dataset identified five clusters, which were annotated based on their gene expression signatures. The “cell of origin” (COO) classification shows two subtypes of DLBCL: the GCB and the ABC DLBCL, which appear to originate from the light zone (LZ) B-cells and cells committed to plasma blast (PBL) differentiation [28]. Our clusterization based on signaling pathway genesets revealed distinct clusters that were not influenced by the different pathological stages, the IPI [29], or the COO classification (Supplementary Figure S1). As shown in Figure 1C, patients tend to be clustered not by the previous classification but by a BCR-related cluster. Only one cluster (C1) contained a high percentage of ABC, and dispersion of the COO cluster and IPI can be seen in other clusters such as C2, C3, C4, and C5. Together, these results clearly demonstrate the existence of BCR signaling-related DLBCL clusters.

Next, we analyzed the cluster-specific expression pattern. When the BCR is activated, it initiates a signaling cascade that results in the activation of the NF-κB, PI3K/mTOR, MAPK, and NFAT pathways. To assess if different pathways were activated in different clusters, we combined GSEA with the conserved marker identification for each cluster (Figure 1D). Conserved markers for each cluster were selected (log_2_ FC threshold = 0.25, adj *p*-values < 0.001), and the top 20 markers were used for downstream analysis (ordering based on average log_2_ FC). The analysis showed highly conserved markers related to the BCR signaling pathway in C1, C4, and C5, while clusters C2 and C3 showed conserved markers related to the JAK-STAT signaling pathway (Figure 1D).

To assess the enrichment of the BCR-related signaling pathways, we conducted log_2_ FC and calculated the percentage of samples expressing each feature for different identity clusters. The C1 cluster was preliminarily identified as an enriched JAK-STAT signaling pathway based on its gene signatures, including high expression of IL-*10RA*, *IL-4R*, and *MYC* (adj-*p* values < 0.0001 and average log_2_ fold change > 0.3). Even so, additional investigation into cell cycling and conserved markers revealed high expression of BCL2 as well as a G2M/S cell cycle state, indicating highly proliferative ABC lymphoma. Cluster C2, which contains patients with different COO clusters, was highly enriched in PI3K-AKT-mTOR-related genes such as *LAMB1*, *LAMA4*, and *LAMC1* (adj-*p* value < 0.0001 and average log_2_ fold change > 0.3), with a positive enrichment score for the JAK-STAT pathway. The C3 cluster is highly enriched for the NF-κB signaling pathway (Normalized Enrichment Score (NES) = 0.8 adj-*p* value < 0.001). Functional enrichment analysis revealed a negative enrichment on the C4 cluster in both NF-κB and JAK-STAT signaling pathways, while conserved marker gene identification showed a significant upregulation of MS4A1, *LTB*, *CD79A,* and *CD79B* (adj-*p* values < 0.0001 and average log_2_ FC > 0.3), suggesting a BCR-dependent signaling pathway. Finally, cluster C5 was highly enriched for the BCR signaling pathway along with other signaling cascades, such as the PI3K-AKT-mTOR, NF-κB, and JAK-STAT pathways. (NES = 0.75 adj-*p* value < 0.001). Briefly, we found different clusters of DLBCL that exhibited distinct BCR-related pathways.

Progression-Free Survival (PFS) rates of patients in different clusters were different from those of patients with COO classification, and PFS rates for classification based on BCR signaling were also different (*p*-value < 0.005). Cluster C4 showed a better PFS with respect to other clusters such as C1 (16 months versus 10 months, *p*-value = 0.0004). This finding was confirmed in our cohort of *n* = 500 patients, which can be clustered based on the BCR signaling pathway (Figure 1E) that might be involved in disease progression and outcome.

### 2.3. DLBCL and pSS Share a Common BCR Signaling Signature

**Figure 2:**
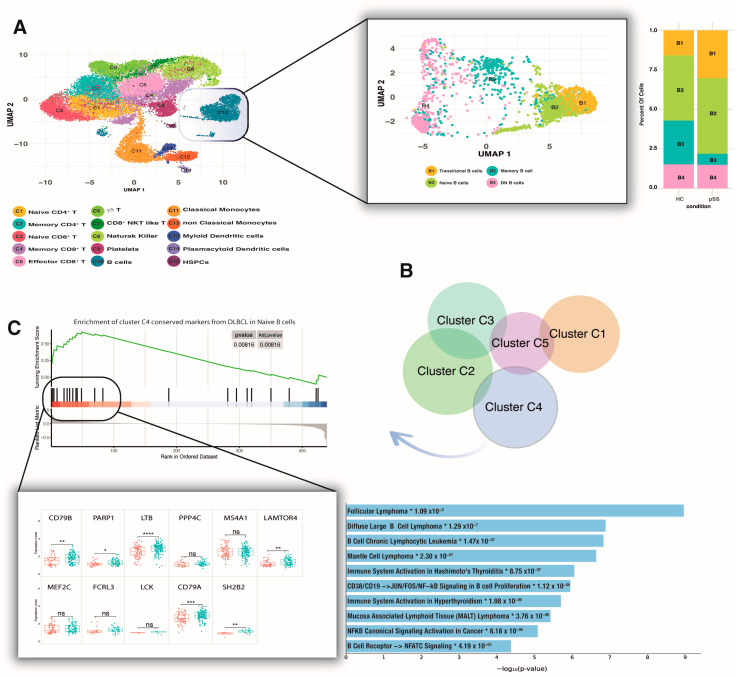
pSS peripheral naive B-cells share the same signature as DLBCL C4. (**A**) High-dimensional reduction of the pSS peripheral transcriptome revealed 15 different clusters. Focusing on B-cells (C10) showed four subpopulations whose frequency was not detected as significant in pSS relative to HC. (**B**) All the DLBCL clusters showed unique markers, and the overlap between clusters was less than 1%. Cluster C4 shared the same signature as pSS peripheral naive B-cells. (**C**) The core of C4 conserved markers enriched in pSS peripheral naive B-cells contains significant overexpression of *CD79B*, *PARP1*, *LTB*, *LAMTOR4*, *SH2B2*, and *CD79A* (ns = not significant; * *p* ≤ 0.05, ** *p* ≤ 0.01, *** *p* ≤ 0.001 **** *p* ≤ 0.0001 (*t*-test)), which is correlated with a different type of B-cell lymphomas such as DLBCL and MALT lymphoma (*log_10_ adj-*p* values < 0.0001). Red box represents HC; green box represents pSS patients. pSS—primary Sjogren syndrome; HC—healthy controls; MALT—mucosa-associated lymphoid tissue.

scRNAseq analysis was performed by the Seurat pipeline on a total of 55,982 cells from 5 healthy controls (HC) (*n* = 26,184) and 5 pSS (*n* = 29,798) [30]. The dimensional reduction was performed using the Uniform Manifold Approximation and Projection (UMAP) algorithm [31], and clusters were identified by the PhenoGraph algorithm [32]. A total of 3048 B-cells were sorted for independent analysis of B-cells from pSS and HC (Figure 2A). Identification of B-cell populations was performed by fully automated and ultra-fast cell-type identification known as ScType [33] based on a reference scRNAseq atlas of peripheral B-cell subsets [34], along with a comprehensive cell marker database as background information. On this ground, we found 4 different B-cell subsets (Transitional “Trans”, Naive, IgM Memory “M-mem”, and IgD^-^ CD27^-^ “DN”) (Figure 2A). We asked whether there is a similarity between the BCR signaling-based clusters previously developed in DLBCL B-cells and B-cells from the peripheral blood and minor salivary glands (SG) of pSS patients and HC.

Briefly, we used a novel cluster similarity approach in which the fold change of B-cell counts for each gene between the pSS and HC groups was computed for the data following normalization and used as input for GSEA. All conserved markers derived from different DLBCL clusters were used as gene sets, and NES > 0.1 and *p*-value < 0.001 were considered cluster similarity. All the clusters showed unique conserved markers, and the overlap between clusters was less than 1% (Figure 2B). Using this geneset database as input for GSEA, we failed to find a significant geneset enrichment for total peripheral B-cells in pSS with respect to HC. At levels of different subsets, we found a significant enrichment of conserved genes from cluster C4 of DLBCL in pSS peripheral naive B-cells (*p*-value < 0.001 and NES > 0.4), but no significant enrichment with other clusters (Figure 2C). GSEA revealed an enrichment core consisting of genes such as *CD79B*, *PARP1*, *LTB*, *PPP4C*, *MS4A1*, *LAMTOR4*, *MEF2C*, *FCRL3*, *LCK*, *CD79A*, and *SH2B2*. We further compared those enrichment core expression levels between pSS and HC subjects and found that only 6 genes (*CD79B*, *PARP1*, *LTB*, *LAMTOR4*, *SH2B2*, and *CD79A*) were significantly overexpressed in naive B-cells derived from pSS subjects (*p*-value < 0.001, Figure 2C). The inferred protein interaction networks were more informative, revealing a strong correlation of those overexpressed genes with different types of B-cell lymphomas such as DLBCL and MALT lymphoma (log_10_ adj-*p* values < 0.0001, Figure 2C).

The A20 protein is an ubiquitin-editing enzyme that plays a central role in the control of nuclear factor κB (NF-kB) activation and is involved both in autoimmunity and lymphomagenesis. Considering that the axis of this protein with its gene (*TNFAIP3*) represents a potent tumor suppressor gene in B-cell lymphoma [35,36], we investigated the measurement of *TNFAIP3* mRNA levels across different B-cell subsets. Our result showed significantly lower *TNFAIP3* expression only in naive B-cells of pSS compared with HC (FC: 0.5 *p*-value < 0.001). Considering that the NF-κ B pathway is not shared by DLBCL and pSS, we did not further investigate this pathway and its inhibitor (Supplementary Figure S2).

Total mRNA extracted from minor SG yielded similar results, with the BCR signaling pathway showing a strong enrichment score and a shared DLBCL cluster signature. Enrichment was conserved at the tissue site for clusters C3 and C4 (*p*-value < 0.001 and NES > 0.4). For deep analysis of the enrichment core, we chose only the top core enrichment of C4, based on rank-order significant results in peripheral naive B-cells in the C4 cluster; we used it for differentially expressed genes (DEG) analysis between conditions and age groups. We divided all pSS salivary gland gene expression profiles into 3 age-related groups: young- (<30 years), middle- (30*–*50 years), and old-aged (>50 years).

**Figure 3:**
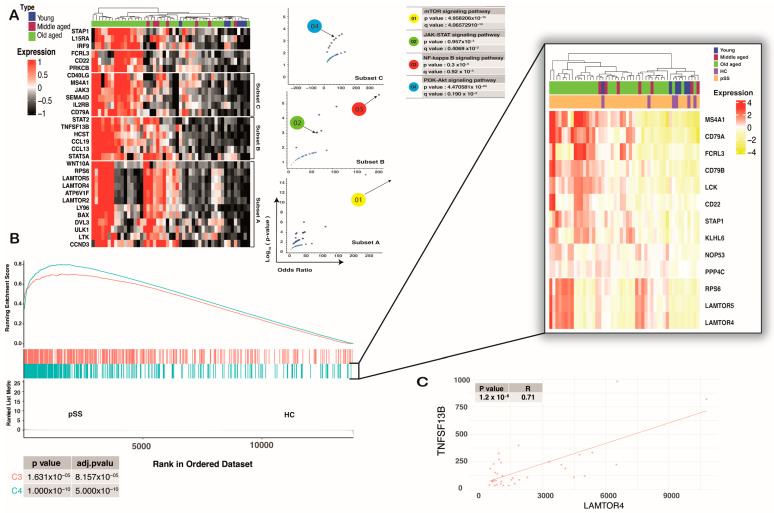
pSS SG-infiltrated cells shared the same signature with C4 of DLBCL. (**A**) Heat map showing expression of BCR signaling-related genes (of total salivary gland RNA) that were significantly up-regulated (fold change ≥ 2 and FDR ≤ 0.05) in the old-aged group when compared to other groups. Those genes tend to be in different subsets based on Euclidean distance. Each of the subsets was correlated with different BCR-related signaling pathways based on the Volcano plot of terms from the Reactome 2022 geneset. Each point represents a term, plotted by its odds ratio (x-position) and −log_10_(*p*-value) (y-position) from the input query gene set enrichment results. (**B**) Conserved markers of C4 show a significant enrichment in SG-infiltrated cells. The core of enrichment displays overexpression of *MS4A1*, *CD79A*, *FCRL3*, *CD79B*, *LCK*, *RPS6*, *LAMTOR5*, *LAMTOR4*, and lesser *CD22*, *STAP1*, *KLHL6*, and *NOP53*. (**C**) Spearman’s rank correlation coefficient on mRNA expression for two genes of interest (*LAMTOR4*, *TNFSF13B*) from only old-aged patients showed a significant positive correlation (*p*-value < 0.0001 considered a significant correlation).

To understand the molecular pathways linked with age in pSS patients, we determined gene expression from whole salivary gland RNA from patients in different age groups. No BCR signaling genes showed a statistically significant expression difference (fold change ≥ 1 and FDR ≤ 0.05) between pSS patients and HC. However, BCR-related differentially expressed genes were found in the old-aged group when compared to other groups. Notably, more than half of all up-regulated genes were associated with various BCR signaling-related pathways that were divided into three subsets, such as subset A (mTOR signaling), subset B (NF-*κ*B signaling), and subset C (PI3K-Akt- signaling) (*p*-value < 0.0001 and q-value < 0.0005) (Figure 3A).

Our study revealed that the enrichment core of the C4 DLBCL cluster was highly expressed in old-aged pSS subjects. CD79A was highly upregulated in old-aged patients (log_10_ FC > 0.4, adjp-*p* value < 0.001), which mirrored BCR signaling in those groups. Similar findings were observed with pSS-naive B-cells at the single-cell level. *LAMTOR4* and *RPS6*, two genes that are conserved markers of cluster C4 related to the mTOR signaling pathway [37], were highly upregulated (log_10_ FC > 0.4, adj-*p* value < 0.001) in pSS SG (Figure 3B).

We then used the Spearman correlation between gene expression and lymphoma development risk to determine the role of those genes in pSS patients. The result showed a strong correlation between *LAMTOR4* and *RPS6* (R > 0.3, *p*-value < 0.001) with *TNFSF13B* expression, suggesting a possible correlation between the increasing activity of the mTOR signaling pathway and DLBCL progression in pSS patients (Figure 3C). In general, it is conceivable that those observations could reflect age-related changes in B-cell infiltration, but a major challenge at the bulk RNA-seq level is that this method cannot reflect changes in immune-cell subsets. However, our research strategy is in line with other already published studies [38,39] that have focused only on gene expression or signal changes based on bulk RNA-seq.

## 3. Discussion

pSS is a B-cell-mediated autoimmune disorder that affects the lacrimal and salivary glands and evolves into NHL in 5% of patients [1]. While pSS has a good prognosis and most patients face relatively few severe difficulties in their daily lives, developing NHL lymphoma could be a challenging issue. Therefore, a helpful strategy to control the progression of pSS is to target either B-cells or find biomarkers involved in the hyperactivation of B-cells. Considering the possibility of progression from pSS to the DLBCL type of NHL [23], we paid attention to cells’ signaling in those two pathologies to find novel biomarkers involved.

Alizadeh et al. [28] have reported two pathologic DLBCL COO clusters (ABC and GCB) based on gene expression profiling. ABC and GCB lymphomas have different signaling patterns, in which BCR and its downstream signaling proteins, NF-κB and PI3K, respectively, play major roles [17]. BCR-mediated signaling not only plays a critical role in the development of DLBCL but also regulates the development of a variety of B-cell-mediated autoimmune disorders, including pSS [40]. Despite multiple attempts to define trial endpoints in pSS patients and rituximab therapy being used for its potential clinical benefits, therapeutic efficacy in pSS remains inadequate [41,42,43]. Thus, our study focused on the role of BCR-mediated signaling in the regulation of cell survival, growth, and apoptosis as a therapeutic target [44,45]. To this end, we constructed a dataset containing BCR, NF-κB, PI3K/mTOR, NFAT, and JAK-STAT (since they cross-talk with NF-κB, [46,47]) pathways. We assessed our clusters based on signaling pathway-related gene expression instead of using gene expression profiling, as performed in studies related to the dataset used by the present study [38,48,49]. We detected five signal-based clusters in DLBCL that were reliant on neither pathological status nor IPI. The clusters’ independence from the pathological condition could help us evaluate the enriched signals in DLBCL and their similarity with pSS clusters. The evaluation of the dataset in peripheral B-cells from pSS patients demonstrated the similarity of significant enrichment of conserved genes between the C4 cluster from DLBCL and pSS peripheral naive B-cells (NES > 0.5, adj-*p* value < 0.01), where the only enriched signal was BCR signaling. The enrichment core contains overexpression of *CD79B*, *PARP1*, *LTB*, *LAMTOR4*, *SH2B2*, and *CD79A*.

The engagement of BCR with cognate antigens leads to constitutive BCR activation. As a result, pSS progresses to lymphoma. CD79a/b are critical components in this cascade, in which ITAM phosphorylation of their cytoplasmic tail is essential to initiate the BCR and downstream signaling [12]. Poly (ADP-ribose) polymerase 1 (PARP1) is an enzyme that functions in DNA damage, repair, transcription, and, through regulation of cellular mitosis and processes such as apoptosis and necrosis, alters the life cycle of cancer cells [50,51]. PARP1 overexpression and PARP1 inhibitors as possible therapies have been reported in numerous cancers [52,53], putting forward PARP1 as a potential biomarker implicated in the development of pSS. Lymphotoxin-β (LTβ) is a type II membrane protein of the TNF family. The major function of LTβ has been identified in the development of tertiary lymphoid organs; these organs appear in pSS patients’ SG and correlate with autoantibody serum levels and, consequently, with the progression of the disease [54]. In non-obese diabetic (NOD) mice, inhibition of LTβ receptor results in the suppression of lymphoid neogenesis in the salivary glands and helps to partially restore their function [55]. The role of active LTβR signaling in lymphocyte migration to lymph nodes makes it a therapeutic approach in inflammatory and infectious diseases, as well as cancer [56]. Moreover, we found the overexpression of *LAMTOR4* by pSS peripheral naive B-cells, which is a component of the regulator complex and participates in the detection of amino acids and the activation of the mTOR pathway [57]. The long-lived and highly proliferating lymphoma B-cells are favored by excessive mTOR pathway activation, which is correlated to poor prognosis in NHL and B-cell acute lymphoblastic leukemia (LAL-B) patients [58]. The upregulation of *CD79A/B* (resulting in BCR hyperactivation), *PARP1* (resulting in increasing cells’ life cycle), and *LAMTOR4* (resulting in survival and proliferation of cells) in pSS naive B-cells could explain why this subpopulation increases in pSS peripheral blood [59]. The overactivation of these genes could affect the response of pSS patients to the therapy. This implies that targeting these genes in pSS may be a helpful strategy for preventing peripheral B-cell activation and migration to SGs, where they can organize ectopic germinal center-like structures, resulting in the symptoms of the disease and its development into lymphoma [60].

Therefore, considering SGs are the main location of activated B lymphocytes that result in pSS symptoms, we also assessed the C4 cluster of DLBCL conserved markers in SG mRNA derived from different pSS patient age groups. Differentially expressed gene analysis showed different subsets of genes that were significantly correlated with BCR-related signaling pathways such as mTOR, JAK-STAT, and PI3K/AKT signaling, indicating that the findings in peripheral blood mirror those in SG. *CD79A/B* and *LAMTOR4* were upregulated in SG. In addition, *MS4A1*, *FCRL3*, *RPS6*, *LAMTOR5*, *LCK*, but to a lesser extent, *CD22*, *STAP1*, *KLHL6*, *NOP53*, and *PPP4C* were also overexpressed mainly in old-aged pSS patients compared to HC. As described above, *CD79A/B* and *LAMTOR4* are essential players in the BCR and mTOR signaling pathways, respectively. Overexpression of membrane-spanning 4-domains A1 (*MS4A1*), also called *CD20*, which is the main marker of B lymphocytes, is consistent with high B-cell infiltration into SG [61]. *FCRL3* encodes a member of the Fc receptor-like glycoproteins, which are predominantly expressed in B-cells and cause aberrant immunological activation and self-tolerance loss [62]. The aberrant expression of *FCRL3* in autoimmune thyroid diseases, Grave’s disease, systemic lupus erythematosus (LES), and pSS has already been noted [62,63], while its exact function in pSS is not clear yet. *RPS6* and *LAMTOR5* are the hallmarks of mTOR signaling, as is *LAMTOR4*. Lymphocyte-specific protein tyrosine kinase (LCK) is a protein tyrosine kinase, with a dual function in BCR activation or inhibition, but its exact function in B-cells is yet unknown [64]. In addition to the above-mentioned genes, lower expression of *CD22* (regulation of the signals implemented by BCR), *STAP1* (BCR downstream-signaling protein 1), and Kelch-like protein 6 (*KLHL6*), which has a role in BCR signal transduction [65], highlights the remarkable role played by BCR in pSS patients’ SGs.

Finally, as reported by Kim et al. [66], overexpression of *CD79B* is sufficient to trigger an alternative signal that drives the unresponsiveness of DLBCL patients to Ibrutinib therapy. Another study by Quartuccio et al. [67] hypothesized that overactivation of BAFF can be the reason for unsuccessful treatment with Rituximab in pSS patients, and our results showed that there is a positive correlation between *LAMTOR4* and BAFF in the pSS SGs, indicating enrichment of mTOR signaling in the pSS SG. It suggests that in addition to BAFF overexpression, excessive BCR signaling and its overexpressed associated genes may also have an impact on the treatment’s efficacy in pSS patients. Thus, we suggest that the overactivation of BCR-related genes such as *CD79A*, *CD79B*, and *LAMTOR4* as the shared overexpressed genes among DLBCL, pSS peripheral, and SGs could be involved in pSS progression into lymphoma and probably be responsible for the failure of Rituximab therapy.

## 4. Materials and Methods

### 4.1. Data Selection and Acquisition

Bulk RNAseq Data acquisition for the present study is fully covered by the TCGA publication guidelines (https://www.cancer.gov/tcga, 16 April 2023). Gene expression data (RNA-seq) of 529 samples and clinical follow-up data with clinicopathological characteristics of patients from DLBCL projects (TCGA-DLBC *n* = 481, NCICCR-DLBCL *n* = 48) were obtained from the Cancer Genome Atlas (TCGA) by using the TCGAbiolinks (version “2.26.0”) [68]. Clinical information, gene expression subtype, and genetic subtype of the DLBCL patients were supplemented by referring to open-access supplementary files of the GDC DLBCL publication [69].

scRNA-seq data of peripheral B-cells from pSS subjects and controls in GSE157278 from the Gene Expression Omnibus (GEO) were used in our study. In GSE157278, peripheral blood mononuclear cells (PBMCs) from 5 pSS subjects and 5 controls were analyzed by scRNA-seq. In addition, a sample with low-quality sequencing was further excluded.

The high-throughput sequencing count dataset, GSE154926, contained 43 pSS and 7 control minor SG samples. For these datasets, the R Bioconductor package DESeq2 (version “1.38.1”) was used for data standardization, to obtain the standardized matrix file, and the “GEOquery” (version “2.66.0”) package in R software was used to download the clinical information. All the information about the sample can be found in the Supplementary Data File).

### 4.2. Gene Set Construction

Firstly, we developed a BCR signaling-related gene set, including 626 genes, by merging gene ontologies, and the Molecular Signatures Database (MSigDB), and KEGG pathways, The initial step yielded 626 unique genes, which were then imported into the ClueGO plug-in of Cytoscape (version “3.9.1”) [70] to find all enrichment terms. The final enrichment result was then sorted based on *p*-value, FDR < 0.0001, and gene size > 10. Only 221 terms containing “Signaling” were selected as the final gene set.

Small (16 terms) and big (221 terms) gene set libraries were used as the backbone for all subsequent analyses.

### 4.3. Bullk RNA Seq Analysis

The training cohort (529 patients with matched normalized RNA-seq data and survival data from TCGA) was used for the construction of BCR-related DLBCL patient clusters based on developed gene set libraries. RNA-seq data from all DLBCL subjects were pre-processed, log_2_-transformed, and analyzed using the DEseq2 R package. BiomaRt (v2.48.2) was used for annotation based on GRCh37/hg19 to cross-map gene symbol identification. The final count matrix contained 59660 FPKM and was projected in the Seurat object, where, after feature selection and scaling of the normalized data, we performed PCA linear dimensional reduction. The first 20 PCs were used to construct the KNN graphic, and the Louvain algorithm was performed for clustering the patients with a resolution parameter set to 0.5. We ran the UMAP method for the non-linear dimensional reduction to visualize the entire dataset.

Cell cycle phases were scored using the list of cell cycle markers from Tirosh et al. that are preloaded in Seurat [71]. Gene set enrichment analysis (GSEA) and circular visualization were used to determine whether scRNA-seq data from bulk GEP can be used to classify individual patients based on the developed BCR signaling gene set. The final patient clustering was used for gene set enrichment, Kaplan–Meier (KM) survival analysis, and to discover the relationships between clinical features and BCR signaling pathway alteration. Eventually, the top 20 conserved markers based on average Log_2_ fold change and adj-pvalue for each cluster were considered the cluster signature.

The R package DESeq2 v1.38.2 [72] was used to perform a differential gene expression analysis of bulk RNA-seq data of minor salivary glands between pSS patients and HC. Normalization of RNA-seq counts data and variance stabilizing transformation (VST) data were used for downstream analysis. We use the default Wald test for differential expression analysis. The method used for adjusting *p*-values was the Benjamini–Hochberg method. Genes with an adjusted *p* value less than 0.05 and an absolute value of fold change greater than 1 were considered DEGs. The logarithmically transformed data were calculated using DESeq2, and the batch effect was removed using the R package limma v3.44.3. After batch effect correction, the data were used for principal component analysis (PCA) and weighted correlation network analysis (WGCNA).

### 4.4. Single-Cell RNAseq Analysis

scRNA-seq analyses were performed using Seurat (Version 4.3.0) and SingleR (Version 2.0.0) [73,74]. Quality control was performed mainly by the number of feature genes and the percentage of mitochondrial gene expression. To characterize the subsets of B-cells precisely, scRNA-seq data of high quality were analyzed. Cells with detected genes above 1000 and a percentage of mitochondrial genes less than 10% were regarded as cells of high quality. Cells were omitted if they were more than 10% of mitochondrial gene expression. Gene counts were normalized with the SCTransform function of Seurat. Intergraded data from all samples was clustered with 15 PCs in combination with the dimensional reduction method of uniform manifold approximation and projection (UMAP). Cell type annotation was performed with ScType [33], and those cells annotated to be B-cells were extracted for subsequent analyses. The feature genes of B-cell subsets were calculated through differential expression analyses in Seurat. A single-cell atlas of peripheral B-cells was used as a reference map to annotate B-cell subtypes [34], and EnrichR was used to analyze disease-related pathways.

### 4.5. Cluster Similarity Using GSEA

Functional annotation of the DEGs was performed with clusterProfiler [75], and gene sets of the DLBCL cluster signature were mainly analyzed in the functional enrichment analyses. Gene sets with adj-*p* values less than 0.05 were considered significantly enriched pathways. A positive enrichment score between the DLBCL cluster signature and the B-cell subset was used to assess cluster similarity.

### 4.6. Statistical Analysis

R software (version 4.0.3) was used to analyze all statistical data. To compare continuous variables between two groups of sample data, the Wilcoxon test was used, while to compare continuous variables among three or more groups of sample data, the Kruskal–Wallis test was used. A test is considered statistically significant at *p* < 0.05. Pearson correlation was used to test the linear correlation between two sets of data, and an absolute Pearson correlation coefficient larger than 0.3 was considered to be correlated. A Pearson correlation is considered statistically significant at FDR < 0.05. We used ggplot2, ggstatsplot, and ggpubr R packages [76,77] for data analysis and visualization.

## Figures and Tables

**Table 1:** The 16 BCR signaling-related pathways and GO signatures.

Name	Description	Accession Code	Number of Gene	Category
D1	B cell receptor signaling pathway	GO:0050853	65	GEO term
D2	negative regulation of B cell receptor signaling pathway	GO:0050859	13	GEO term
D3	positive regulation of B cell receptor signaling pathway	GO:0050861	14	GEO term
D4	phosphatidylinositol 3 kinase signaling (PI3K)	GO:0014065	48	GEO term
D5	PI3K Akt signaling pathway	GO:0043491	55	GEO term
D6	Activated NTRK2 signals through PI3K	M27910	7	KEGG pathway
D7	NIK/NF kappa B signaling	GO:0038061	43	GEO term
D8	NF kappa B signaling pathway	hsa04064	105	KEGG pathway
D9	PI3K activity	GO:0035004	18	GEO term
D10	positive regulation of PI3K activity	GO:0043552	38	GEO term
D11	negative regulation of PI3K activity	GO:0043553	10	GEO term
D12	positive regulation of PI3K signaling	GO:0014068	87	GEO term
D13	negative regulation of PI3K signaling	GO:0014067	20	GEO term
D14	mTOR signaling pathway	hsa04150	157	KEGG pathway
D15	JAK STAT signaling pathway	hsa04630	167	KEGG pathway
D16	regulation of JAK STAT cascade	GO:0046425	12	GEO term

## Data Availability

All data used for this study are available as Supplemental Materials.

## Supplementary Materials

The following supporting information can be downloaded at: https://www.mdpi.com/article/10.3390/ijms24098385/s1.
