# Supplementary material for: B-Cell Receptor Signaling Is Thought to Be a Bridge between Primary Sjogren Syndrome and Diffuse Large B-Cell Lymphoma
Source: Int J Mol Sci. 2023 May 7;24(9):8385. doi: 10.3390/ijms24098385 (PMC10179133; doi:10.3390/ijms24098385)
Supplement: Supplementary file 1 [file ijms-24-08385-s001.zip › figures.pdf]

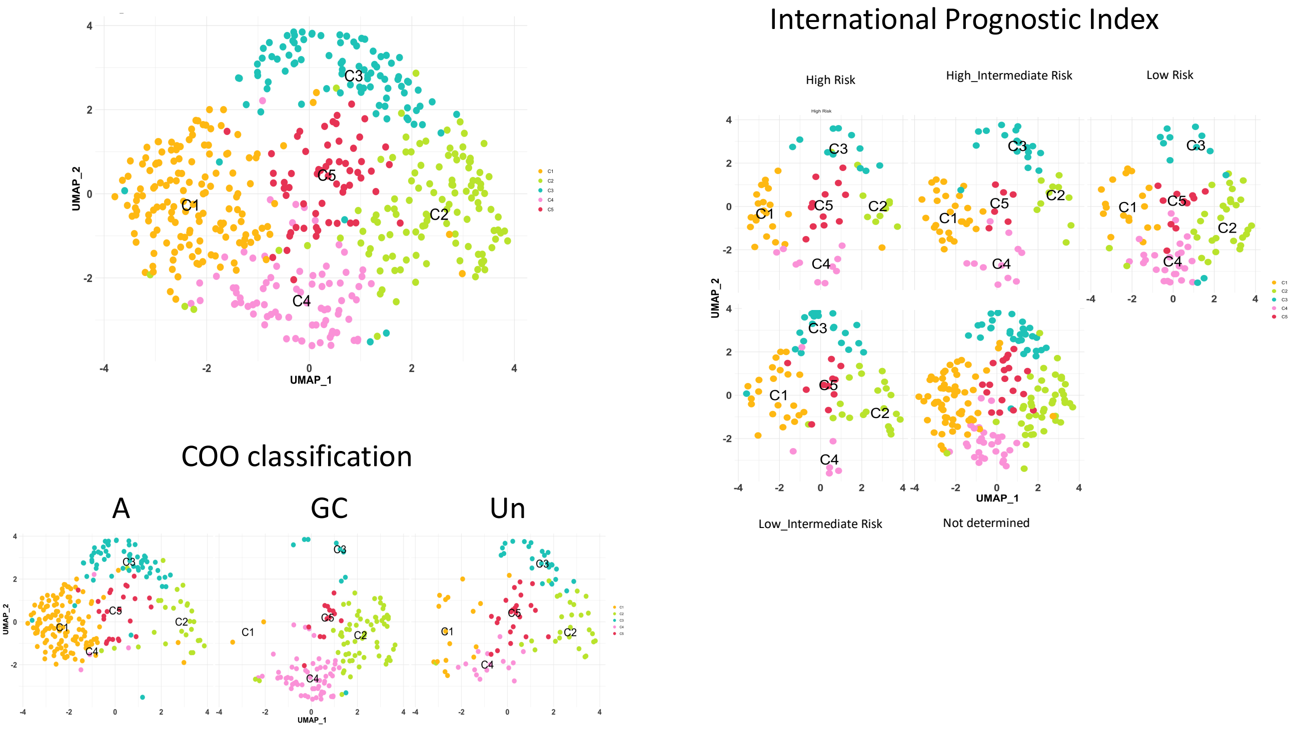

Supplementary Figure S1: UMAP plots of final dimensional reduction clearly show that there are no pathological conditions specific cluster, indicating that these clusters are independent from any batch effect has no effect on dimensional reduction and clusterization.

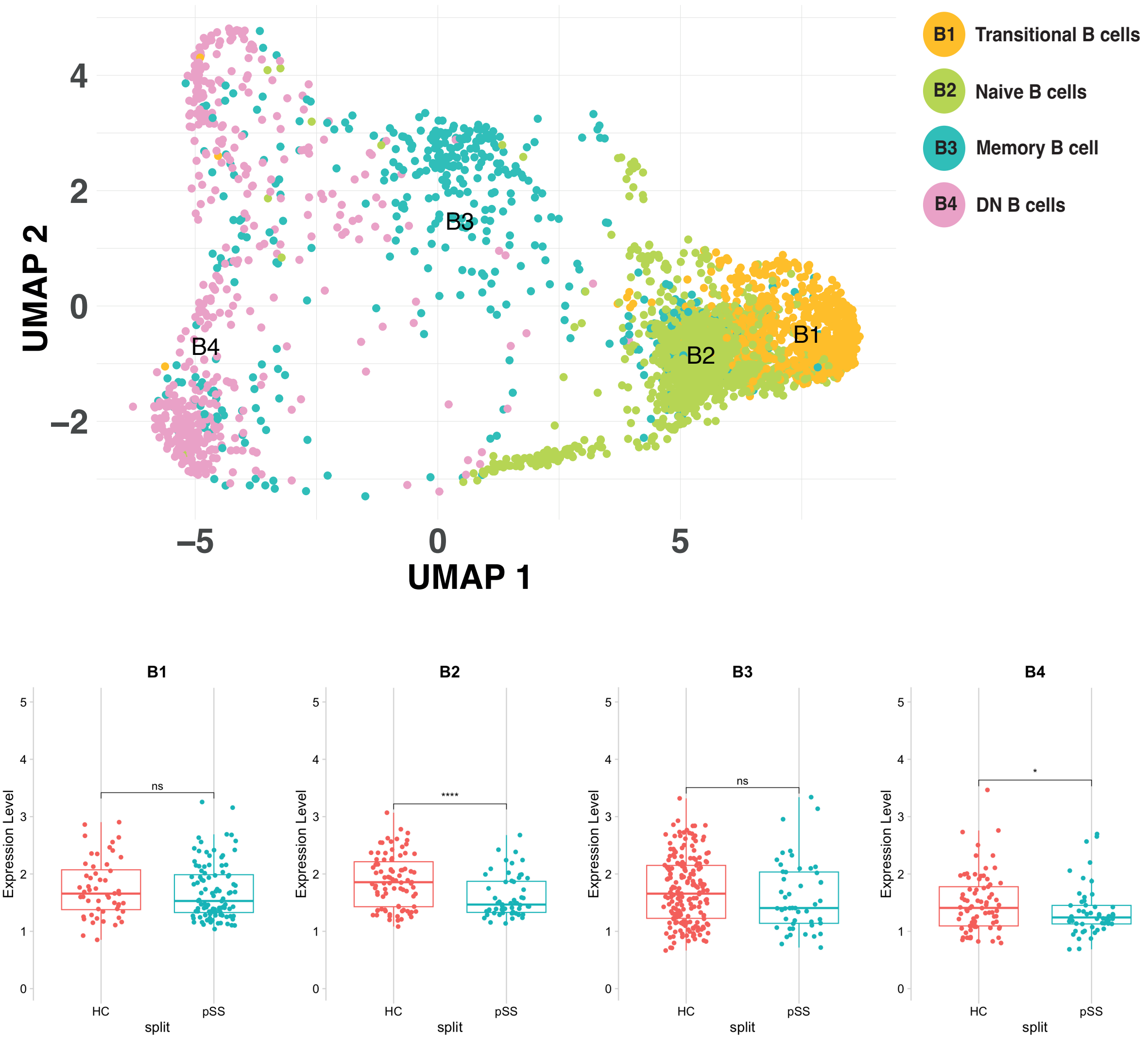

Supplementary Figure S2: Differentially expression of *TNFAIP3* gene in B-cell subsets of pSS compared to HC.
